# An Exceptional Case of Blow-Out Fracture with Complete Globe Dislocation into the Maxillary Sinus: Diagnostic Imaging and Surgical Reconstruction

**DOI:** 10.3390/diagnostics15212705

**Published:** 2025-10-25

**Authors:** Krzysztof Gąsiorowski, Michał Gontarz, Jakub Bargiel, Tomasz Marecik, Grażyna Wyszyńska-Pawelec

**Affiliations:** Department of Cranio-Maxillofacial Surgery, Jagiellonian University Medical College, 30-688 Cracow, Poland

**Keywords:** orbital fracture, blow-out fracture, orbital reconstruction, orbital imaging

## Abstract

Orbital floor fractures are primarily caused by blunt trauma to the area around the eyes. These injuries most commonly affect the orbital floor and medial wall due to the fragility of these structures. The mechanism typically involves transmission of force through the orbital rim or an acute increase in intraorbital pressure caused by globe displacement. Blowout fractures often occur alongside additional maxillofacial fractures and periorbital soft tissue injuries. The reported causes mirror those of general maxillofacial trauma and include motor vehicle collisions, interpersonal violence, falls, sports-related injuries, incidents involving firearms, and occupational accidents. Here, we present the case of a 56-year-old male patient who sustained an exceptionally rare injury pattern characterized by a complete orbital floor fracture with globe dislocation into the maxillary sinus. Such extensive fractures are associated with significant functional impairments, including diplopia, enophthalmos, and restricted extraocular muscle movement, as well as marked aesthetic deformity. Comprehensive diagnostic imaging, comprising coronal, sagittal, and three-dimensional CT reconstructions, was crucial for accurately assessing the extent of bony disruption and soft tissue involvement. Particular emphasis should be placed on imaging that clearly delineates the extraocular muscles and the optic nerve, as precise evaluation of these structures is essential for surgical planning and prognosis. Surgical management involved repositioning of the globe and the orbital contents, followed by reconstruction of the orbital floor using a titanium mesh anchored to the infraorbital rim. This case highlights the technical challenges of total orbital floor reconstruction, emphasizing the importance of meticulous anatomical restoration for achieving optimal functional and aesthetic outcomes.

**Figure 1:**
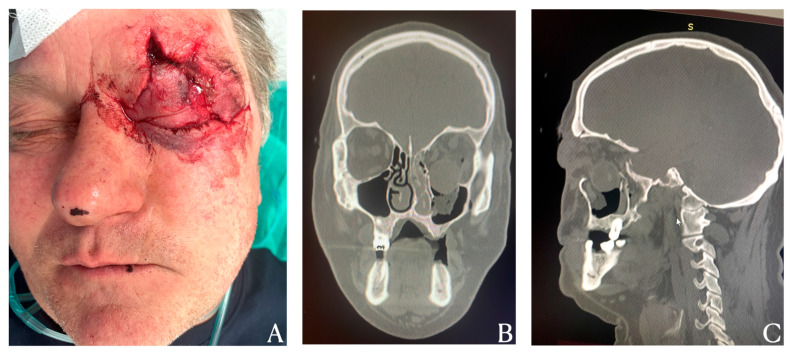
A 56-year-old male patient was admitted to the Department of Cranio-Maxillofacial Surgery at the University Hospital in Kraków on an emergency basis following blunt facial trauma as shown in Figure 1. Clinical examination revealed a displaced blowout fracture of the left orbital floor, accompanied by dislocation of the globe into the maxillary sinus and contused lacerations of the upper eyelid and frontal region. The injury occurred earlier that day when the patient was struck by a wooden block while operating a circular saw. Upon admission, the patient was fully conscious and able to recall the circumstances of the accident. Initial management at a district hospital included radiological assessment of the head and neck, ophthalmologic consultation, and the administration of tetanus prophylaxis. The patient was then transferred to a tertiary care center for specialized treatment. Upon examination, lacerations to the eyelid and frontal area were noted (**A**). Computed tomography (CT) scans in the coronal and sagittal planes revealed a complete fracture of the orbital floor, with the globe displaced downwards into the maxillary sinus and the extraocular muscles and optic nerve stretched (**B**,**C**) [1,2]. Orbital floor fractures most frequently result from blunt trauma to the periocular region and typically affect the orbital floor and medial wall, the most fragile components of the orbit. The injury mechanism generally involves either the transmission of force through the orbital rim or a sudden increase in intraorbital pressure caused by displacement of the globe. Blowout fractures are often associated with additional maxillofacial fractures and periorbital soft-tissue injuries. The most common causes are similar to those seen in general maxillofacial trauma and include motor vehicle collisions, interpersonal violence, falls, sports-related injuries, firearm incidents, and occupational accidents [3]. While orbital floor fractures are relatively common, complete disruption of the orbital floor with herniation of the globe into the maxillary sinus is an extremely rare and severe injury. Such cases pose substantial diagnostic and surgical challenges, with a high risk of functional sequelae including diplopia, enophthalmos, and restricted extraocular movement, as well as significant aesthetic deformity [4]. A comprehensive CT evaluation, including coronal, sagittal, and three-dimensional reconstructions, is essential for accurately assessing the extent of bony disruption and soft-tissue involvement. Particular attention should be given to the condition of the extraocular muscles and optic nerve, as visualization of these structures is critical for surgical planning and prognostic assessment.

**Figure 2:**
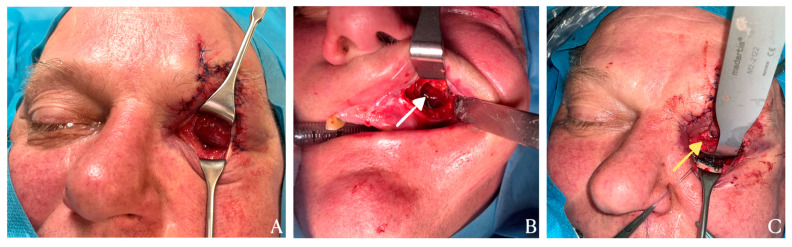
Intraoperatively, an empty left orbital cavity with exposed periorbital fat was exposed, confirming that the globe had been completely dislocated into the maxillary sinus as shown in Figure 2. (**A**). The surgical procedure began with exposure of the anterior wall of the left maxillary sinus via an incision in the vestibular fornix. The anterior wall of the sinus was then carefully opened and elevated using a piezoelectric saw, providing access to the sinus cavity and direct visualization of the displaced globe (**white arrow**). The displaced globe was gently elevated along the fractured orbital floor fragments and repositioned into the orbital cavity, ensuring preservation of the extraocular muscles and optic nerve (**B**). To maintain the globe in the correct position during reconstruction, a catheter was placed in the maxillary sinus beneath the bony fragments to provide temporary support. A transconjunctival approach was then used to expose the infraorbital rim and fractured orbital floor. After the orbital contents were meticulously repositioned (**yellow arrow**), the orbital floor was reconstructed using a precontoured titanium mesh, which was securely fixed to the infraorbital rim with two titanium screws (**C**). This provided stable structural support, restoring the integrity of the orbital floor and preventing postoperative enophthalmos. The contused laceration of the upper eyelid was repaired using reconstructive techniques analogous to those applied in eyelid reconstruction following the excision of malignant skin tumors, ensuring proper restoration of eyelid anatomy and function [5].This surgical approach is consistent with contemporary principles of orbital floor reconstruction, which emphasize precise anatomical restoration while minimizing the risks of enophthalmos, diplopia, and extraocular muscle restriction. Previous studies have highlighted that both optimal surgical timing and accurate anatomical reconstruction are crucial determinants of functional and aesthetic outcomes in orbital blowout fractures—particularly among pediatric and adolescent patients, in whom long-term orbital growth may be affected [6]. These observations further validate our approach and reinforce the importance of individualized treatment planning and stable, three-dimensional orbital reconstruction to restore normal ocular alignment and orbital volume [7,8].

**Figure 3:**
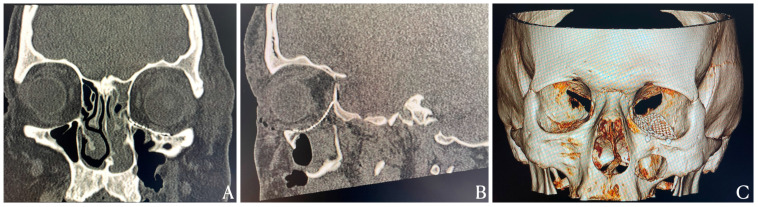
Postoperative imaging confirmed successful reconstruction of the orbital floor using a titanium mesh implant. As shown in Figure 3 coronal CT scans revealed (**A**) symmetrical restoration of orbital floor continuity, with partial reconstruction of the medial wall. Sagittal views showed re-establishment of orbital floor integrity extending posteriorly to the bony ledge (**B**). Three-dimensional CT reconstructions provided a comprehensive view of the restored orbital anatomy, confirming accurate implant alignment and positioning (**C**). The primary objectives of orbital floor reconstruction are to restore the integrity of the orbital walls, re-establish ocular motility, and prevent delayed enophthalmos, thereby optimizing both functional and aesthetic outcomes. A wide range of materials can be utilized for orbital reconstruction, including autogenous grafts harvested from the anterior wall of the maxillary sinus, iliac crest, or auricular and nasal septal cartilage. Among alloplastic materials, titanium mesh remains the most commonly employed non-resorbable implant due to its excellent biocompatibility, high mechanical stability, and intraoperative malleability, which allow for precise replication of the native orbital contour [9]. A recent systematic review and meta-analysis by Kotecha et al. reported a trend toward improved postoperative orbital volume restoration, reduced enophthalmos, and shorter operative times with the use of patient-specific implants (PSIs) compared with conventional techniques. However, these differences did not reach statistical significance, suggesting that current evidence does not yet confirm the superiority of PSIs. Consequently, the selection of the reconstructive material should remain at the surgeon’s discretion, guided by clinical context and intraoperative findings [10].

## Data Availability

Restrictions apply to the availability of these data. Data was obtained from patients treated at the Department of Cranio-Maxillofacial Surgery, Cracow, Poland, and cannot be shared, in accordance with the General Data Protection Regulation (EU) 2016/679.
